# The Complex Emergency Database: A Global Repository of Small-Scale Surveys on Nutrition, Health and Mortality

**DOI:** 10.1371/journal.pone.0109022

**Published:** 2014-10-21

**Authors:** Chiara Altare, Debarati Guha-Sapir

**Affiliations:** Centre for Research on the Epidemiology of Disaster, Institute of Health and Society, Université catholique de Louvain, Brussels, Belgium; University of the Stellenbosch, South Africa

## Abstract

Evidence has become central for humanitarian decision making, as it is now commonly agreed that aid must be provided solely in proportion to the needs and on the basis of needs assessments. Still, reliable epidemiological data from conflict-affected communities are difficult to acquire in time for effective decisions, as existing health information systems progressively lose functionality with the onset of conflicts. In the last decade, health and nutrition humanitarian agencies have made substantial progress in collecting quality data using small-scale surveys. In 2002, a group of academics, non-governmental organizations, and UN agencies launched the Standardized Monitoring and Assessment of Relief and Transitions (SMART) methodology. Since then, field agencies have conducted thousands of surveys. Although the contribution of each survey by itself is limited by its small sample and the impossibility to extrapolate results to national level, their aggregation can provide a more stable view of both trends and distributions in a larger region. The Complex Emergency Database (CEDAT) was set up in order to make best use of the collective force of these surveys. Functioning as a central repository, it can provide valuable information on trends and patterns of mortality and nutrition indicators from conflict-affected communities. Given their high spatial resolution and their high frequency, CEDAT data can complement official statistics in between nationwide surveys. They also provide information of the displacement status of the measured population, pointing out vulnerabilities. CEDAT is hosted at the Centre for Research on the Epidemiology of Disasters, University of Louvain. It runs on voluntary agreements between the survey implementer and the CEDAT team. To date, it contains 3309 surveys from 51 countries, and is a unique repository of such data.

## Introduction

Evidence has become central for humanitarian decision making, as it is now commonly agreed that aid must be provided solely in proportion to the needs and on the basis of needs assessments [1] [2] [3]. Still, reliable epidemiological data from conflict-affected communities are difficult to acquire in time for effective decisions. Existing health information systems progressively lose functionality with the onset of conflict, reducing their usefulness as data sources. Demographic and Health Surveys (DHS) and Multiple Indicator Cluster Surveys (MICS) are typically alternative sources for reliable data. However, population in conflict affected regions within the country are frequently excluded from the survey due to insecurity. Furthermore, the intervals at which these surveys are conducted are relatively lengthy (e.g. five years) while the health status of communities living in insecurity tends to change rapidly. Nonetheless, the soundness of their methodology and their regularity make them valuable sources for baselines and long-term projections, but less useful for shorter-term humanitarian aid decisions.

In the last decade, health and nutrition humanitarian agencies have made substantial progress in collecting quality data using small-scale surveys. In 2002, a group of academics, nongovernmental organizations, and UN agencies launched the Standardized Monitoring and Assessment of Relief and Transitions (SMART) methodology [4] and the associated “Emergency Nutrition Assessment” (ENA) software. It consists of a cluster sampling method to collect comparable data on nutrition and mortality. Now widely used by relief agencies, this method generates quality epidemiological data to quantify crisis thresholds, monitor effectiveness and strengthen evidence-based response [5].

In the recent years, field agencies have conducted thousands of surveys, increasingly using the SMART methodology. Although the contribution of each survey by itself is limited by its small sample and the impossibility to extrapolate results to national level, their aggregation can provide a more stable view of both trends and distributions in a larger region. The Complex Emergency Database (CEDAT) was set up in order to make best use of the collective force of these surveys. Functioning as a central repository of nutrition and mortality surveys from conflict affected areas, it can provide valuable information on trends and patterns of mortality and nutrition indicators on communities affected by complex emergencies [6] [7] [8]. To date, it is a unique repository of such data.

The Centre for Research on the Epidemiology of Disasters (CRED) at the Catholic University of Louvain has developed and run CEDAT since its inception. CEDAT data is available at the project website (www.cedat.be) subject to the confidentiality clearance from the data source.

## Methods

### Organisation

The CEDAT database forms the nucleus of the conflict research programme of CRED. It is managed by a small team of researchers, interns and has IT support from CRED.

The initiative is guided by two committees. A Technical Advisory Group (TAG) provides direction for the overall project, inputs for priority setting and feedback on the relevance of the products. The members are technical experts from universities, donor organisations, NGOs and UN. In addition to the TAG, a small Expert Group was established to provide detailed technical guidance to the CEDAT team for issues identified by the TAG. Its members are A. Colombo, P. Spiegel, O. Bilukha, M. van Herp, J. Pedersen, and R. Garfield.

### Data sources

A network of field agencies regularly contributes surveys to CEDAT. The group includes UN agencies, country clusters, non-governmental organisations, Ministries of Health. As this is a voluntary effort, it has taken time to establish a two-way relationship of trust and confidence where both parties (the CEDAT team and the survey producer) benefit from mutual services. Based on this history, Memoranda of Understanding specifying the nature of collaboration have also been signed with principle survey providers (Action Contre la Faim International, Concern Worldwide, GOAL, International Medical corps, International Rescue Committee, Merlin, Tearfund, World Vision International). In addition to the contributed surveys, the CEDAT team also extract data from surveys reported in peer-reviewed journals.

### Inclusion/exclusion criteria

The surveys must meet the following criteria to be included in the database:

Defined population based sampling frame. Data from health institutions are not included.
Probabilistic sampling procedure with minimum required sample size to ensure representativeness of the results. Surveillance reports as well as data collected using convenience sampling are not included.
Use of standard anthropometric techniques that follow internationally recognised methods and tools recommended by WHO and UNICEF.
Presentation of results expressed in prevalence of Global Acute Malnutrition, Crude Mortality Rate, Measles Containing Vaccine coverage.
Survey setting recognised as a complex emergency defined as follows:
  CEDAT includes crises characterized by extreme vulnerability which display the following features: a) Government is unwilling or incapable to effectively respond, resulting in a need for external assistance; b) political oppression or armed conflict; c) displacement; d) increased mortality [9].

### Database content

The CEDAT Expert Group identified five indicators to assess the severity of a crisis. These indicators are:

Crude Mortality Rate (CMR): The number of deaths over a given period of time divided by an estimate of the population at risk of dying during that period. Mortality rates from emergency settings are most commonly reported as deaths/10,000/day. Other reporting formats such as deaths/1,000/day or month can be transformed to allow comparison.
Under 5 Mortality Rate (U5MR): Although in these surveys the U5MR is conceptually the same as the CMR above (number of deaths over the number of children in the age group), this is not the probability of survival as defined in demography.
Global Acute Malnutrition (GAM): weight-for-height (WFH) index <−2 standard deviations from the median weight of the reference distribution for children of the same height, and/or having oedema. GAM is entered in CEDAT as prevalence expressed as z-scores or percentage of the median, presented in relation to NCHS reference and WHO standard population.
Severe Acute Malnutrition (SAM): weight-for-height index <−3 standard deviation from the median weight of the reference distribution for children of the same age. CEDAT enters this information in the same format as for GAM.
Measles Containing Vaccine coverage (MCV): number of children vaccinated against measles over the children in the appropriate age group eligible for vaccination. It is expressed as prevalence.

For each indicator the point estimate, confidence interval and sample size are recorded.

Data is available at the database website: www.cedat.be, which is publically available.

Indicators are extracted from survey report and all information is anonymous.

### Additional information

Besides the above mentioned core indicators, survey reports record chronic malnutrition, underweight, Mid-Upper Arm Circumference and adult malnutrition, with variable frequency. As enrolment to nutrition programmes is based on MUAC, it is increasingly reported and entered into CEDAT.

In addition, following information are extracted from survey reports:

**Location**: country; first, second and third administrative level; city; camp.
**Time**: start and end date of the data collection;
**Population group**: survey population is defined according to its displacement status; resident (people affected by a conflict who remained in their habitual place of residence); internally displaced persons (those who have been forced to leave their habitual residence without crossing an international border, in order to avoid the effects of armed conflict [10]), refugee (any person who has crossed an international border to seek refuge from violence (UN 1950)); returnee (persons who voluntarily returned after being forced to displace), nomad (pastoral populations whose livelihoods are compromised by civil conflict. Moving about in search of water and pasture for their herd, they do not have a stable residence).
Surveyed area population: the **sampling frame** of the survey usually drawn from sources such as Census, WFP.
**Methodology**: sampling design, sample size, population size, mortality recall period.
**Cause of deaths**: proportion of deaths due to violence and due to diseases.
Name of **organisation** conducting the survey.

### Data quality control

Data quality control is an essential component of the survey validation process. The CEDAT team checks following aspects before entering the survey results:

Survey completeness: the CEDAT team and the EG have defined a list of elements that need to be in the report in order for this to be considered complete. A completeness checklist has been developed and agreed upon by partner NGOs.
Data inconsistencies: for example, different sample sizes recorded or dissimilarities between information in the executive summary and in the main report;
Errors: for example, number of deaths in the total population smaller than deaths among children, wrong age category for MCV coverage (6 to 59 months instead of 9 to 59).

All surveys that require clarifications are classified as pending until they can be validated. Surveys that do not receive clearance for public use are kept confidential and not made available to the public until approval is obtained. These confidential surveys therefore can only be included for aggregated display. The team typically maintains close contact with the survey providers in the field for clarifications and additional information.

### Data workflow

**Figure 1:**
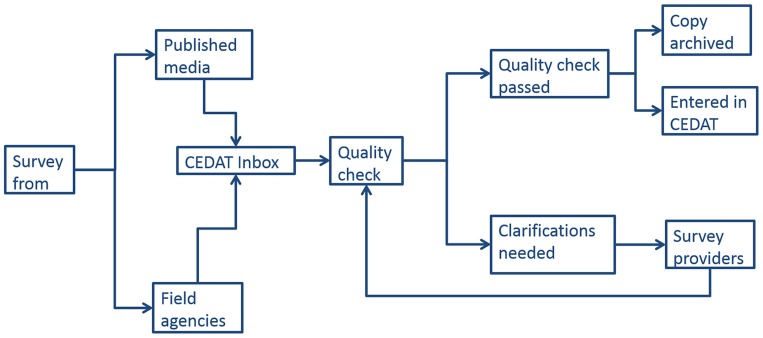
CEDAT data flow.

Figure 1 describes the data workflow of the database. Once a survey report is received, its completeness and methods are reviewed as described above. Either clarifications are required and the survey provider is contacted, or the survey is validated and entered in the database. A hard copy of the report is archived.

### Applications of the data

**Figure 2:**
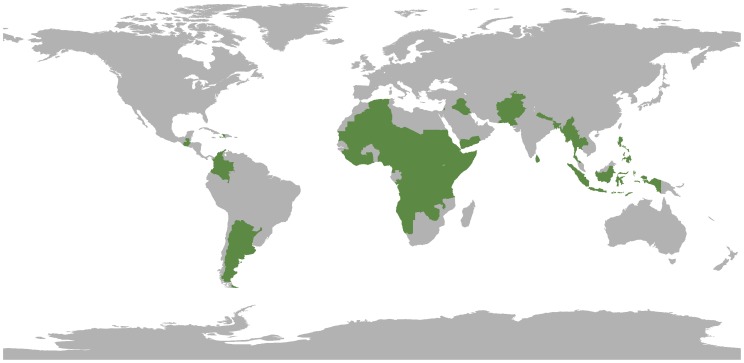
CEDAT countries.

CEDAT has recorded 3,309 surveys as of December 2013, from 51 countries covering a period 2000–2013 (Figure 2). In total, it includes 24,889 data points for nutrition, 4,502 for mortality and 3,652 for vaccination coverage. Table 1 shows the top ten countries by number of surveys available.

**Table 1:** CEDAT top ten countries by number of surveys available.

Country	Number of surveys
Sudan	636
Democratic Republic of the Congo	461
Somalia	371
Ethiopia	369
Kenya	131
Uganda	125
Niger	121
Angola	91
Chad	91
Malawi	77

As a unique repository of surveys from conflict affected population, the CEDAT database allows for a rich variety of analysis.

**Figure 3:**
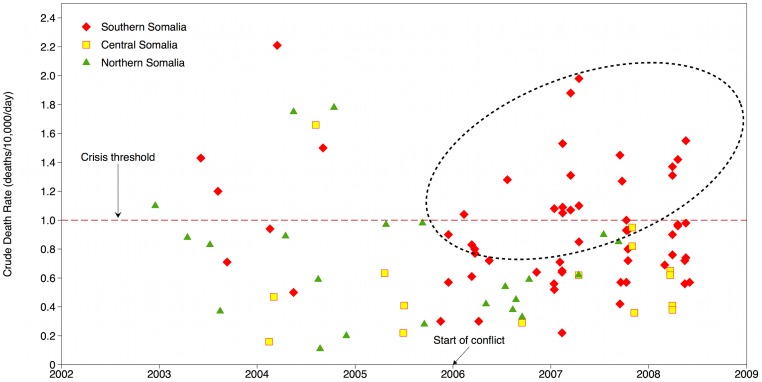
Crude death rates in Somalia, 2002–2008. Note: *Note: Reprinted from* [*6*] *under CC-BY license*.

First, trends and distributions of mortality and nutrition indicators can be explored using survey results over time and space. For example, the human impact and the location of the intense fighting in early 2006 in Somalia is fully reflected in the increased mortality rates in the conflict-affected South (red points in Figure 3) [6]. In contrast, the increasingly more stable situation in Ethiopia is revealed in the decreasing child death rates over the last decade [11].

**Figure 4:**
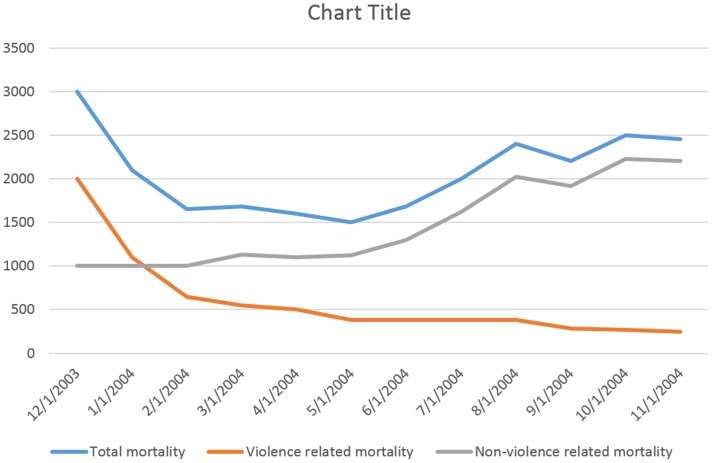
Mortality patterns in Darfur, 2003–2004. *Note: Adapted from* [8].

Second, patterns and causes of deaths can also be investigated. Causes of deaths are an extremely important aspect for preventive and response action. For example, the analysis of mortality surveys in Darfur showed that mortality rates were driven by non-violent deaths [8], indicating that the majority of fatalities in conflict are not directly related to violence, but are due to the collapse of the health system (Figure 4). Despite the limitations inherent to the identification of the cause of death by non-medical staff, this information is crucial to understand the impact of conflict on the population, and to best prepare the response.

**Figure 5:**
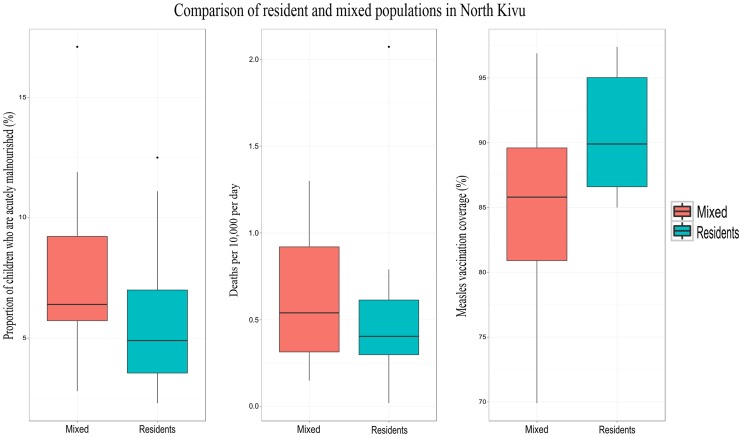
Mortality, malnutrition and vaccination coverage in North Kivu: comparison between residents and population groups with displacement experience (IDP or returnee), 2010–2012. *Note: Reprinted from* [*12*] *under CC-BY license, with permission from CRED, original copyright 2012*.

Third, an essential criterion for identifying vulnerable groups is their displacement status. Violence often forces communities to seek refuge elsewhere, and the setting in which they relocate has major consequences on their health status. For instance in Darfur, surveys of populations with large proportions of internally displaced people reported higher mortality rates than those among only non-displaced individuals. However, the effect of displacement on mortality varies when differentiating between violence-related and non-violence-related deaths. Among IDP, mortality associated with violence is generally lower than mortality due to indirect causes [8]. In North Kivu, groups with previous displacement experience (IDPs or returnees) fare worse than resident as far as mortality, nutrition and vaccination coverage is concerned [12] (Figure 5).

Fourth, CEDAT data combined with population figures provide estimates of affected children at higher spatial resolution [13]. This provides information on the caseload by district, which is crucial not only for funding appeals but also for targeting resources.

**Figure 6:**
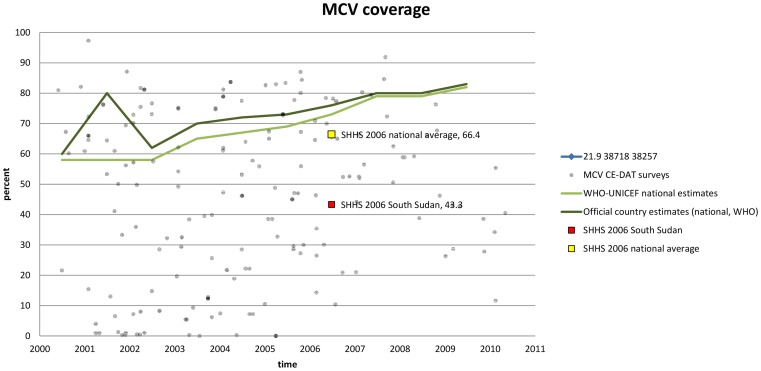
Measles Containing Vaccine coverage in southern Sudanese states (2000–2011). *Note: Reprinted from* [*14*] *under CC-BY license, with permission from CRED, original copyright 2011*.

Fifth, timely data can complement official statistics in-between nationwide surveys and provide a refined picture of the situation. For example, data from South Sudan show that heterogeneity in measles vaccination coverage is high, and that national average masks important geographical differences. Such information is of evident relevance for policy definition (Figure 6) [14].

Finally, the analysis of hundreds of small-scale surveys can provide insight into methodological issues: data quality from emergencies [15] [16]; usefulness of nutrition indicators in identifying emergencies [7]; appropriateness of established thresholds [7]. It can also highlight areas requiring further capacity building in epidemiological skills. While data collection methodology has advanced over the years, result interpretation could still be improved.

## Discussion

Some of the results presented above point to the potential policy implications of aggregated analysis of small-scale surveys.

Data at high resolution (spatial or temporal) and disaggregated by population groups are useful to target vulnerable groups. Knowing who is in need and their location enhances aid effectiveness, as targeting improves. Small-scale surveys go beyond national average estimates that may mask inequalities, and highlight differences within countries. As we have seen, displacement has different consequences on health status, and this has to be considered in the response.

Aggregating small-scale surveys increases the return on investment incurred by the data provider. These data collection exercises are rather costly, not only in monetary terms, but especially in terms of human resources and opportunity costs. Unfortunately, only the main results are used for programme monitoring and advocacy, while further analysis is rarely undertaken. Additional investments are necessary to strengthen analysis capacities and use of existing information [17]. Statistical techniques such as meta-analysis and Bayes LQAS [18] can exploit CEDAT data further and provide important insights to decision makers.

The SMART's software ENA has helped significantly in bringing about a tangible improvement in the quality of both data and reporting. Survey providers increasingly use its plausibility checks during data collection (and not only at the end), and can therefore better monitor data quality “on the job”. They also include results from the plausibility check in the final report, providing an indication of the reliability of the data. In addition, ENA creates a report template and tables, therefore increasing standardisation in reports and reducing “copy-paste” errors. Much of the progress has been in the nutrition component, where global and local experts were able to come together for cohesive and coordinated way forward. Progress still remains to be achieved for the mortality and food security components.

The CEDAT effort has however several limitations.

Data quality is a matter of concern, as validity checking by the CRED staff are limited to only the information available in the survey reports. Ideally, results can be more accurately evaluated if they are examined against the raw data. As the CEDAT project was not set up to receive and process raw datasets, it has therefore be unable to undertake this particular step although this could have been possible.

Being a voluntary initiative, only surveys shared by partners are entered in the database. Estimating CEDAT coverage is a challenge, as no centralised list of implemented surveys exists. However, the establishment of the humanitarian cluster system has contributed to coordinate information on surveys and will facilitate a better assessment of CEDAT coverage. In fact, the country nutrition clusters often keep track of the nutrition surveys conducted by their partners. This can support the CEDAT team in the identification of surveys.

The selection of core indicators current included in CEDAT needs review. The indicator list could be expanded or modified to better include current practices such as the addition of MUAC measures based on its increased use.

In addition, the nutrition transition is already occurring in many low-income countries and overweight is becoming a public health issue. The extent to which this could affect communities living in long term insecurity should be assessed. Initial evidence from protracted emergency settings exists [19], but further studies are necessary.

## Conclusions

The CEDAT repository remains an important source of health and nutrition surveys from conflict affected populations. The potential for policy settings and monitoring of trends can be further exploited and strengthen evidence based decision making in hard to reach populations.
